# Supplementary material for: Telehealth Delivery of a Multi-Disciplinary Rehabilitation Programme for Upper Gastro-Intestinal Cancer: ReStOre@Home Feasibility Study
Source: Cancers (Basel). 2022 May 30;14(11):2707. doi: 10.3390/cancers14112707 (PMC9179413; doi:10.3390/cancers14112707)
Supplement: Supplementary file 1 [file cancers-14-02707-s001.zip › cancers-1693923-supplementary.pdf]

cancers MDPI

Supplementary file 1. Sample Interview Guide

ReStOre@Home–Sample Interview Guide

Post Programme Semi-Structured Interview Questions

- Interview data related to feasibility will be reported in feasibility manuscript
- All interview data will be analysed for detailed qualitative manuscript

Questions related to exercise programme, education & dietetics input

Did you find this programme beneficial?

If so, how? If not, why not?

- Has the programme helped with your **recovery** from your cancer treatment?

  If so, how? If not, why not?

- Has the programme impacted your **fitness**?

  If so, how? Which aspects of the programme helped improve your fitness? (If improved)

- Did the programme influence your **eating and nutrition**?

  Have your nutrition-related symptoms changed since starting the programme? E.g. nausea, appetite, dumping syndrome

- Has the programme changed how you manage your **daily activities and routines?**

  If so, how? If not, why not? (additional activities, routines, activity levels)

- Has the programme impacted your **mental health and well-being**?

  If so, how?

- Have your energy levels / has your level of fatigue changed since starting ReStOre@Home? (Depending on pre-treatment symptoms)
- Have your **family and/or friends** noticed any difference since you participated in the programme?

  If so, how?

+ prompts for particular components of programme: education, dietetics, exercise if not mentionned

Structure of programme

What are you overall impressions of the programme? What did you enjoy/not enjoy?

What is your opinion on the structure of the programme: the length, the content, the schedule?

Is there anything you would change to make the programme better in the future? (education, nutrition and physical activity components)

Telehealth questions

What is your opinion on doing ReStOre@Home online?

What were the benefits of doing it online? And the down sides?

Was doing ReStOre@Home online easier or more difficult than you imagined? How so?

What did you think of the Salaso system?

What did you like about it?

Can you recommend any changes which would make this system better?

What is your opinion of the heart rate monitor watches?

Did you find them helpful? If so, how? If not, why?

Do you have any other feedback about ReStOre@Home that you would like to give?

Supplementary Table S1. Physical and anthropometric outcomes of the ReStOre@Home feasibility study, n=9.

**Measure**	**T0 Mean (SD)**	**T1 Mean (SD)**
Physical outcomes		
CPET Peak power (watts), mean (SD)	123.33 (36.4)	128.33 (43.23)
Grip strength right (kg), mean (SD)	31.02 (10.2)	33.9 (9.4)
Grip strength left (kg), mean (SD)	29.2 (12.4)	30.78 (9.9)
Leg press (kg), mean (SD)	88.45 (22.1)	93 (28.6)
SPPB total, mean (SD)	10.78 (1.72)	10.78 (1.86)
SPPB balance	3.89 (0.33)	3.56 (1.33)
SPPB gait speed	4.00 (0.0)	3.89 (0.33)
SPPB chair stand	2.89 (1.45)	3.33 (1.41)
**Godin-Shephard Leisure-Time Physical Activity Questionnaire**		
Physical activity type	**T0 Mean (SD)**	**T1 Mean (SD)**
Strenuous	0.0 (0.0)	5.0 (15.0)
Moderate	11.11 (12.94)	19.44 (14.67)
Mild	14.0 (12.64)	10.0 (9.6)
Total	25.11 (19.16)	34.44 (16.88)
**Anthropometric measurements (n=9)**	**T0 Mean (SD)**	**T1 Mean (SD)**
Weight (kg)	74.46 (13.59)	72.45 (14.12)
BMI (kg/m2)	25.60(4.31)	24.83(4.42)
Waist circumference (cm)	95.25(12.22)	93.97(13.59)
Middle arm circumference (cm)	28.83(2.82)	28.51(2.74)
**Bioimpedance analysis (n = 8)**	**T0 Mean (SD)**	**T1 Mean (SD)**
Fat mass (kg)	19.92(10.91)	18.40(11.32)
Fat mass (%)	24.87(8.77)	23.37(9.33)
Fat free mass (kg)	55.45(3.91)	55.73(3.50)

Fat mass index (kg/m2)	6.60(3.33)	6.23(3.69)
Fat free mass index (kg/m2)	18.65(1.70)	18.76(1.67)
Skeletal muscle mass (kg)	27.28(2.28)	26.96(2.35)

CPET: Cardiopulmonary Exercise Testing; SD: Standard Deviation; SPPB: Short Physical Performance Battery test; BMI: Body Mass Index.

Supplementary Table S2. Quality of life outcomes of the ReStOre@Home feasibility study, n=9.

	**T0 Mean (SD)**	**T1 Mean (SD)**
**Multidimensional Fatigue Index**		
General Fatigue	10.38 (2.39)	10 (2.2)
Physical Fatigue	12.72 (1.49)	13.75 (3.11)
Mental Fatigue	11.63 (2.07)	11.38 (2.62)
Reduced Anxiety	12.88 (1.55)	12.75 (2.19)
Reduced Motivation	12.88 (3.14)	13.25 (1.58)
**EORTC QLQ-C30: functional scores**	**T0 Mean (SD)**	**T1 Mean (SD)**
Global health status/QoL	77.78 (16.67)	71.30 19.59)
Physical functioning	82.96 (17.36)	88.15 (15.19)
Role functioning	75.93 (25.15)	79.63 (2.6)
Emotional functioning	83.33 (23.94)	77.78 (32.0)
Cognitive functioning	83.33 (27.64)	79.63 (34.13)
Social functioning	62.96 (28.6)	74.07 23.73)
**EORTC QLQ-C30: Symptom Scales**	**T0 Mean (SD)**	**T1 Mean (SD)**
Fatigue	32.10 (21.83)	33.33 (26.64)
Nausea & vomiting	20.37 (30.93)	20.37 (27.36)
Pain	20.37 (23.24)	25.93 31.3)
Dyspnoea	22.22 (23.57)	22.22 23.57)
Insomnia	22.22 (23.57)	37.04 35.14)
Appetite loss	33.33 (40.82)	18.52 (33.79)
Constipation	14.81 (24.22)	0.00 (0.0)
Diarrhoea	40.74 (32.39)	29.63 (20.03)
Financial difficulties	25.93 (26.43)	18.52 (24.22)
**EORTC QLQ-OG25***	**T0 Mean (SD)**	**T1 Mean (SD)**
Dysphagia	18.06 (25.85)	11.11 (27.22)
Eating restriction	26.04 (31.63)	17.71 (31.95)
Reflux	22.92 (21.71)	25.00 (35.63)
Odynophagia	12.50 (23.15)	18.75 (35.0)
Pain and discomfort	18.75 (22.6)	31.25 (35.0)
Anxiety	27.08 (26.63)	31.25 (30.13)
Eating with others	20.83 (35.36)	8.33 (15.43)
Dry mouth	12.50 (35.36)	16.67 (35.63)
Trouble with taste	16.67 (25.2)	8.33 (23.57)
Body image	8.33 (15.43)	8.33 15.43)
Trouble swallowing saliva	12.50 24.8)	8.33 (15.43)
Choked when swallowing	12.50 (24.8)	0.00 (0.0)
Trouble with coughing	16.67 (25.2)	20.83 (24.8)
Trouble talking	16.67 (25.2)	0.00 (0.0)

Weight loss	12.50 (17.25)	16.67 (17.82)
Hair loss	33.33 (57.74)	0.00 (0.0)

*n=8 as questionnaire not completed by one participant.

Supplementary Table S3. Nutrition-related symptoms.

	T0 Mean (SD)	T1 Mean (SD)
**GSRS***		
Abdominal Pain (score range 1-7)	2.37 (1.56)	2.25 (1.46)
Reflux syndrome (score range 1-7)	2.05 (1.48)	1.88 (1.43)
Diarrhoea syndrome (score range1-7)	2.7 (1.51)	2.66 (1.62)
Indigestion syndrome (score range 1-7)	2.63 (1.45)	2.8 (1.67)
Constipation syndrome (score range 1-7)	1.59 (0.61)	1.66 (0.74)
Total score (score range 5-35)	11.36 (5.19)	11.28 (6.14)
**SNAQ****		
Total score (score range 4-20)	13.56 (3.21)	14.56 (3.36)
SNAQ ≤14 No (%)	5(55.5 %)	3 (33.33)

* scores are calculated by taking the mean of the items completed within an individual scale, with higher scores indicating greater severity of symptom; ** SNAQ score ≤ 14 indicates significant risk of at least 5% weight loss within six months.
